# Who is Who in *Carbon Balance and Management *2006

**DOI:** 10.1186/1750-0680-2-1

**Published:** 2007-01-22

**Authors:** Georgii A Alexandrov

**Affiliations:** 1 Institute of Atmospheric Physics, Russian Academy of Sciences, Pyzhevsky 3, Moscow, Russia

## Abstract

This editorial provides a subject index from published articles, active researchers, and published papers in the field of carbon balance and management.

## Background

Developing a policy relevant understanding of the global carbon cycle requires a high degree of interdisciplinarity. Therefore, research published in *Carbon Balance and Management *involves cutting across disciplines and consulting with specialists in various fields. This editorial is to support an interdisciplinary effort with relevant bibliographic information [see Additional file 1] including a source of names to consult with and a list of subjects that may appear in the papers. Terms may differ depending on context.

## Subject index

This list of words and wordings [see Additional file 2], selected from the articles published in the first volume of the journal, represents the phraseology of carbon cycle science. It was compiled to provide help in searching relevant web resources. This list is also presented below together with links to the articles where a given subject is treated or mentioned.

ability to retain organic carbon [14]

acceptable climate change [6]

acid neutralizing capacity of seawater [4]

acid-base balance [2]

acid-base imbalances in marine organisms [2]

activity data [9]

actual country-specific information [9]

adverse conditions [6]

afforestation [15,134]

agriculture sector [9]

alkalinity [2]

allocation of carbon [6]

anomalies in atmospheric CO_2 _increase [7]

anomalous CO_2 _flux [7]

anomalously extreme climate [6]

Anthropocene [3,4]

anthropogenic emissions [7]

Asia region [9]

atmosphere-ice-ocean carbon cycle model [2]

atmospheric CO_2 _concentrations [2,5,88]

average surface temperature [2]

baseline energy consumption from air-conditioning [12]

baseline value for SOC [14]

biologically mediated pH changes [2]

biosphere simulation model [6]

burnt biomass [7]

calcareous shells [2]

capacity to absorb anthropogenic CO_2 _[5]

capacity to act as sinks [3]

carbon budgets [7,11,85,133]

carbon conserving practices [4]

carbon cycle feedbacks [3,42,44,49,50,81]

carbon emissions [8,11]

carbon fertilisation [6,53,55]

carbon flows [8]

carbon flux anomalies [7]

carbon fluxes [3,7,8,17,46,55,65]

Carbon Management Education [13]

carbon price incentive schemes [15]

carbon sequestration [4,15,107,134,135]

carbon sink [5,45,73]

carbon stocks in forest biomass [15]

carbon tax [15]

carbon uptake [5-7,15,36,49]

carelessness feedback [4]

cation exchange capacity [14]

change of vegetation type [6]

changes in the moisture regime [3]

changes in weather patterns [6]

Cities for Climate Change Program [11]

climate change [2,6,14,18,40,43,44,46,49,58,60,81,108,134,136]

climate change feedbacks on the carbon chemistry [2]

climate policy [5]

climate scenario [6]

CO_2 _biological pump [2]

CO_2 _emissions in the commercial sector [12]

CO_2 _uptake by the ocean [2,34]

collapse of the Amazonian rain forest [6]

compensating effects [2,3]

compensatory mechanisms [6]

consumption activities [9]

conversion of natural lands [8]

country-specific emission factors [9]

coupled atmosphere-ocean mode [2]

cover fraction of major vegetation types [6]

current forest cover [15]

data set [7,8,34,63,133]

dead organic matter [8]

decline in boreal forest area [6]

decline in forest area [6]

decrease in pH due to ocean warming [2]

decrease of biomass [6]

decreases in transpiration [6]

decreasing rainfall [6]

default activity data [9]

default emission factors [9]

deforestation [6,15,134]

deforestation emissions [15]

deforestation tax [15]

desertification [14]

DIC concentrations [2]

direct anthropogenic emission of CO_2 _[8]

direct effects of ocean warming [2]

direct human influence [5]

direct injection of carbon into the deep ocean [4]

dissolution of exoskeletal components [2]

disturbance in terrestrial ecosystems [3]

disturbances of the global carbon cycle [7]

drought-induced tree line [6]

Dynamic Global Vegetation Models [6,43,48,61,70]

dynamics of terrestrial ecosystems [3]

ecological modernization [11]

ecosystem physiology [3]

eddy covariance measurements [7]

EEZ [5]

EEZ carbon sink [5]

effect of income [11]

effects of temperature and precipitation [7]

embedded carbon [11]

emission factors [9,12]

emission reduction targets [12]

emission sources [9,105,106]

emissions reduction target [11]

emitting mechanisms from sources [9]

energy consumption in typical offices [12]

energy savings potential [12]

energy usage within buildings [12]

energy use [11]

enhanced litter production [6]

enhanced plant growth [3]

environmental regulations [11]

environmental stress [6]

estimation methods [9]

exceptionally dry years [6]

Exclusive Economic Zone [5]

expanding land use [6]

extended dry seasons [7]

extent of ocean acidification [2]

feedbacks and non-linearities [8]

feedbacks and nonlinearities [3]

feedbacks between climate and vegetation [6]

financial mechanisms [15]

fire control in forests [4]

forest [3,6,14,15,17,51,54,55,57,75,79,119,133]

forest degradation [15]

forest expansion [15]

fossil fuel consumption [4]

fuelwood production [15]

full carbon budgets for cities [11]

functional strategies [6]

geoengineering strategies [4]

geographical pattern of vegetation [6]

geologic sequestration [4]

GHG inventories [9]

global carbon trading [5]

global decline in SOC [14]

greenhouse gas emission scenario [6]

greenhouse gas emissions [11]

greenhouse gas inventories [9,12]

guidelines of the Intergovernmental Panel on Climate Change [9]

heat stress on boreal trees [6]

heating, ventilating, and air conditioning [12]

heterotrophic respiration [7]

holistic view of the carbon cycle [3]

human emissions-atmospheric CO_2 _equation [3]

human well-being [6]

HVAC [12]

impact of climate change [6,14]

incentives for keeping the forest carbon stock intact [15]

increase in fire frequency [6]

increase in oceanic [2]

increased deciduousness [6]

increased water demand [6]

increased water use efficiency [6]

increases in growing stock [15]

increasing concentration of carbonic acid [3]

increasing greenhouse gas concentrations [6]

industrial carbon dioxide emissions [11]

industrialization [11]

information exchange activities [9]

injected carbon [4]

insensitivity of pH to climate change [2]

institutional drivers [11]

institutional impacts [11]

intergenerational equity [4]

invariant allocation of carbon gains [6]

IPAT [11,104]

IPCC guidelines [9]

Kuznets curve [11]

Kyoto Protocol [4,5,134]

land ecosystems [6]

land use change [7]

land use model [15]

Land Use, Land-Use Change and Forestry [9]

land-use change [11]

large-scale climate anomalies [7]

leaf nitrogen contents [6]

living biomass [8]

locally appropriate levers for carbon management [13]

locally derived regulatory oversight [11]

long term means to store CO_2 _[4]

lower wet season precipitation [7]

magnitude of carbon fertilisation effects [6]

marine carbon cycle [3]

marine organisms [2]

meridional change in pH [2]

Miami model [14]

mitigating the heat load of buildings [12]

mitigation and adaptation policies [6]

mobilisation of nitrogen [3]

modernization effect [11]

Montreal Protocol [9]

national carbon accounts [5]

national communications [9]

national greenhouse gas inventories [11]

NEE anomalies [7]

net carbon exchange [6]

net carbon loss [6]

net climate change feedback [2]

net ecosystem production [15,133]

net loss of carbon [3]

net primary production [7,14,71,72]

net primary productivity [71,116]

net sink of carbon [6]

neutralizing capacity of calcium carbonate [4]

non-woody vegetation [6]

northward expansion of the boreal forest [6]

NPP [7,8,14]

nutrient and water constraints [6]

nutrient limitations of vegetation growth [6]

ocean acidification [2,19]

ocean pH [2,20]

ocean warming [2]

ocean warming feedback [2]

oceanic anthropogenic CO_2 _sink [5]

oceanic CO_2 _uptake [2,18,67]

oceanic uptake of anthropogenic CO_2 _[2]

past changes in climate [6]

permafrost soils [3]

permafrost thawing depth [6]

pest outbreaks [3]

pH of seawater [2]

phosphorus availability [14]

pools of carbon [3]

population growth [11,92]

purposeful carbon sequestration [4]

purposeful sequestration [4]

quantifiable mitigation strategies [14]

rate of acidification [2]

rates of leakage [4]

recession of the boreal forest [6]

reduce energy consumption in the HVAC [12]

reduced precipitation [6]

reduced soil respiration [7]

reducing deforestation [15]

reduction in DIC growth [2]

regional carbon budget [11]

regional changes in vegetation structure [6]

reporting country inventory practices [9]

reporting requirements [9]

reservoir to purposefully sequester carbon [4]

response of marine organisms to pH changes [2]

response of vegetation [7]

responses of plant functional types to climate [6]

responses to a warming climate [3]

retention of forests [15]

rising acidity of the ocean [3]

robust assessment [6]

salinity [2]

saturation state of calcium carbonate [3]

sea-ice extent [2]

sectoral groups of energy [9]

sensitivity of future oceanic acidification [2]

sensitivity of heterotrophic respiration to soil moisture [7]

sensitivity to natural disturbances [6]

sequestration efficiency [4]

shifts in spatial rainfall distribution [7]

short-lived plant parts [6]

slow down climate change [6]

SOC [14]

socioeconomic drivers [6]

soil crusting and compaction [14]

soil microbial activity [7]

soil organic carbon [14,108,109,112,115,130]

soil organic matter [7,14,112,114]

soil processes [7]

soil respiration [3,6,7,16,84]

soil survey [14]

solubility induced changes [2]

spatial distribution of vegetation [6]

speciation of carbon within the ocean [2]

species composition [6]

steady state analysis [14]

technological efficiency [11]

temporal development of vegetation [6]

temporal variability of heterotrophic respiration [7]

terrestrial balances of carbon [6]

terrestrial carbon balance [6]

terrestrial carbon cycling [14]

terrestrial carbon sink [6]

terrestrial feedback on climate [6]

terrestrial modulation of atmospheric carbon anomalies [7]

terrestrial vegetation [6,52]

the impact on soil respiration [3]

the lack of actual country-specific information [9]

the value of forest land [15]

thermodynamic equilibration [2]

transfer of CO_2 _from the atmosphere to the oceans [4]

transition from temperate savannah to subtropical woodland [6]

trophic structure of marine ecosystems [3]

uncertainty of estimated emissions [9]

UNFCCC [9]

UNFCCC requirements [9]

Urban and Regional Carbon Management [13]

urban areas [11]

urban territories [8,102]

urban vegetation [8]

urbanised ecosystem [8]

urbanized areas [11]

value of the regional network [9]

variations in the CO_2 _growth rate [7]

voluntary environmental management standard [11]

vulnerable to rising temperatures [3]

water-limitation of NPP [7]

weakening oceanic sink [3]

WGIA [9]

wildfires [3]

woody encroachment [6]

world-wide cooling [7]

## Author index

The list of names cited in the first volume of the journal provides some information about the research community involved in the study of the global carbon cycle either directly or indirectly. This information is intended for those who are considering *Carbon Balance and Management *as a medium for conveying their findings and evaluating whether they would be of sufficiently immediate interest to researchers in the broad range of disciplines associated with the studies of the global carbon cycle.

Acock, B. [128]

Adams, A.F.R. [112]

Adams, D.E. [131]

Aerts, K. [25]

Alcamo, J. [41]

Alder, J. [41]

Alexandrov, G. [135]

Alexandrov, G.A. [133,10]

Amato, M. [123,132]

Anderson, B. [80]

Andren, O. [122]

Angert, A. [74]

Apps, M.J. [17]

Arellano, A. [66]

Arneth, A. [70]

Asner, G.P. [71]

Asshoff, R. [55]

Aumont, O. [19]

Aumont, O.L. [67]

Bala, G. [50]

Baldocchi, D.D. [75]

Banks, H.T. [64]

Barrett, D.J. [116]

Barry, J.P. [24]

Bazilevich, N.I. [89]

Beaufort, L. [25]

Benitez, P.C. [134,135]

Benthien, A. [25]

Berry, J.A. [83,82]

Berthelot, M. [46]

Betts, R. [50]

Betts, R.A. [57,42,51,43]

Bignucolo, O. [55]

Biraud, S. [74]

Birdsey, R. [71]

Boden, T.A. [75]

Bondeau, A. [43,70]

Bonfils, C. [74]

Bopp, L. [50,67,19]

Bousquet, P. [67,76,65]

Boutin, J. [36]

Brewer, P.G. [24]

Brovkin, V. [43,50]

Buck, K.R. [24]

Buermann, W. [74,80,76]

Bullister, J.L. [34]

Cadule, P. [50]

Caldeira, K. [20]

Campbell, J. [16]

Canadell, J. [129]

Canan, P. [13]

Catton, W.R. [91]

Chou, L. [25]

Ciais, P. [46,67,65]

Coleman, K. [120]

Collatz, G. [66]

Collatz, G.J. [83,77]

Collins, M. [51]

Colunga-Garcia, M. [108]

Cooper, C. [64]

Cornelissen, J.H.C. [16]

Cox, P. [50]

Cox, P.M. [57,42,51,44]

Cramer, J.C. [92]

Cramer, W. [41,43,56,76,6,47,70]

Crill, P. [79]

Cure, J.D. [128]

da Rocha, H.R. [79]

Dala, O.E. [129]

Dale, V.H. [110]

Daube, B.C. [79]

Davidson, E.A. [81]

Davis, M. [78]

de Camargo, P.B. [79]

de Freitas, H.C. [79]

DeAngelis, D.L. [110]

DeFries, R. [86]

DeFries, R.S. [77]

Delille, B. [25]

Diamond, L. [94]

Dickinson, R.E. [80,1]

Dickson, A.G. [39]

Dietz, T. [95,104]

Dix, M.R. [31]

Doney, S. [50]

Doney, S.C. [19]

Drapek, R.J. [59]

Dufresne, J.L. [46,44]

Duncan, O.D. [96]

Dunlap, R.E. [91]

Eby, M. [50]

Ehrlich, P. [98]

Eichhout, B. [60]

Ellsworth, D.S. [54]

Emanuel, W.R. [110]

Engel, A. [25]

Erbrecht, T. [7,6]

Erlinger, J.R. [129]

Ewert, F. [118]

Fabry, V.J. [19]

Farquhar, G.D. [82,88]

Fasham, M.J.R. [88]

Feely, R.A. [19]

Field, C. [71]

Fisher, V. [43]

Foley, J.A. [43,85,136]

Francey, R. [67]

Friedlingstein, P. [46,44,50,76,65]

Friend, A.D. [43]

Fung, I. [74,50]

Gage, S.H. [109,108]

Garcia, H.E. [35]

Gattuso, J.P. [25]

GCTE, N.E.W.S. [16]

Genovese, V. [73]

Gerber, S. [61,49]

Gerten, D. [52,56,47,87]

Gifford, R.M. [117]

Giglio, L. [77,66]

Gnanadesikan, A. [19]

Gordon, C. [64]

Gordon, H.B. [30]

Goulden, M.L. [88,79]

Grace, P.R. [109,14,108,132]

Grant, R.F. [125]

Gregory, J.M. [64]

Gruber, N. [36,19,65]

Gu, L. [75]

Gurevitch, J. [16]

Haberlandt, U. [52]

Hanaoka, T. [106]

Hansen, J. [68]

Hansen, M. [86]

Hansen, P.J. [27,28]

Harlay, J. [25]

Harris, P.P. [57,51]

Hart, J.L. [93]

Hartley, A.E. [16]

Hashimoto, H. [72]

Hasselmann, K. [49]

Hattenschwiler, S. [55]

Haugen-Korzyra, K.L. [125]

Hausman, J.A. [97]

Heemann, C. [25]

Heimann, M. [45,67,88]

Hennessy, K. [14]

Heyder, U. [6]

Hicke, J.A. [71]

Hickler, T. [56,78]

Hiederer, R. [118,119]

Hiley, J.C. [125]

Hirst, A.C. [30,18]

Hoffmann, L. [25]

Hogg, E.H. [58]

Holdren, J.P. [98]

Holland, E. [71]

Hooss, G. [49]

Houghton, R.A. [45]

House, J.I. [45]

Hulme, M. [63]

Huntingford, C. [57,51]

Hutyra, L. [79]

Ikaga, T. [105]

Isbell, R.F. [114]

Ishida, A. [19]

Ishimatsu, A. [26]

Izaurralde, R.C. [125]

Jackson, R.B. [129]

Jacquet, S. [25]

Jans, D.C. [125]

Janssens, I.A. [81]

Jaramillo, V.J. [88]

Jenkins, J.C. [71]

Jenkinson, D.S. [120,130,131]

John, J. [50]

Johns, T.C. [64]

Jolly, W.M. [72]

Jones, C. [50]

Jones, C.D. [57,42,51]

Jones, P.D. [63]

Jones, R. [119]

Jones, R.J.A. [118]

Joos, F. [50,61,49,19]

Kainuma, M. [106]

Kankaanpaa, S. [119]

Kaplan, J.O. [70]

Karl, T.R. [40]

Kasibhatla, P. [77,66]

Kasischke, E. [66]

Kasischke, E.S. [77]

Kato, S. [105]

Kato, T. [50]

Katterer, T. [122]

Kawamiya, M. [50]

Keel, S.G. [55]

Keeling, C.D. [67,72]

Keeling, R.F. [35,67]

Keller, M. [79]

Kern, J.S. [115]

Key, R.M. [34,36,19]

Kheshgi, H. [67]

Kheshgi, H.S. [88]

Kikkawa, T. [26]

Kindermann, G. [15]

King, A.W. [124,110]

Kirchhoff, V. [79]

Kita, J. [26]

Klooster, S. [73]

Knorr, W. [50]

Koch, G.W. [54]

Kolp, P. [106]

Korner, C. [55]

Kraxner, F. [135]

Kubiske, M.E. [54]

Kucharik, C. [43]

Kuhnz, L. [24]

Kumar, V. [73]

Kurz, W.A. [17]

Ladd, J.N. [123,109,132]

Langenbuch, M. [29]

Le Quere, C. [67,36,88]

Lebel, L. [99]

Leemans, R. [60]

Levis, S. [70]

Lieth, H. [126]

Lindner, M. [119]

Lindsay, A.M. [111]

Lindsay, K. [50,19]

Lloyd, J. [84]

Lomas, M.R. [43,48]

London, B. [100]

Los, S. [71]

Lovera, C.F. [24]

Lucht, W. [80,7,52,56,76,6,47,70,87]

Luo, Y.Q. [53]

Maier-Reimer, E. [36,19]

Marchetti, C. [32]

Marion, G.M. [16]

Masui, T. [41]

Matear, R. [2,19]

Matear, R.J. [18,34]

Matross, D.M. [79]

Matsumoto, K. [4]

Matthews, H.D. [50]

McCallum, I. [15,135]

McGill, W.B. [125]

McKinley, G. [65]

McNeil, B.I. [34,2,5]

McPhaden, J. [69]

McSweeney, K. [136]

Menton, M. [79]

Meyer, J. [119]

Meyer, R. [49]

Michalsky, J.J. [75]

Mikolajewicz, U. [36]

Miller, J.B. [77]

Miller, S.D. [79]

Millero, F.J. [39]

Milligan, C.L. [21]

Mitchell, J.F.B. [64]

Mitchell, M.J. [16]

Monfray, P. [46,36,19]

Montanarella, L. [118,119]

Mooney, H.A. [129]

Moorcroft, P.R. [62]

Mouchet, A. [19]

Munger, J.W. [75,79]

Murakami, S. [105]

Myneni, R.B. [80,76,72,73]

Najjar, R.G. [19]

Nakicenovic, N. [106]

Naumburg, E.S. [54]

Neilson, R.P. [59]

Nejstgaard, J. [25]

Nemani, R.R. [72]

New, M.G. [63]

Nix, H.A. [113]

Norby, R.J. [53,16]

Norman, J. [136]

Obersteiner, M. [134,15,135]

Ofarrell, S.P. [30,31]

Oikawa, T. [133]

Olsen, S. [66]

Orr, J.C. [36,19]

Pacala, S. [33]

Palmer, J. [36]

Parton, W.J. [121]

Pathe, C. [87]

Pedersen, M.F. [27,28]

Pelez-Riedl, S. [55]

Peltzer, E.T. [24]

Peng, T.H. [110]

Pepin, S. [55]

Peylin, P. [67,65]

Piper, S.C. [67,72]

Pittock, A.B. [113]

Pizay, M.D. [25]

Plattner, G.K. [49,19]

Polglase, P.J. [127]

Pomaz, V.L. [102]

Portner, H.O. [29]

Post, W.M. [14,124,110]

Potter, C.S. [80,73]

Prentice, I.C. [43,61,45,49,67,76,88,47,70]

Probert, M.E. [114]

Pyle, E.H. [79]

Quere, C.L. [65]

Raddatz, T. [50]

Ramankutty, N. [43,45,136]

Rametsteiner, E. [15]

Randerson, J. [66]

Randerson, J.T. [71,77]

Rasmussen, P.E. [121]

Rayner, P. [44,50,65]

Rayner, P.J. [67]

Reginster, I. [118,119]

Reich, P.B. [54]

Reick, C. [50]

Reipschlager, A. [29]

Reynolds, R. [68]

Riahi, K. [106,135]

Ribas-Carbo, M. [83]

Rice, A.H. [79]

Riebesell, U. [25]

Ringler, D.C. [41]

Robertson, G.P. [109,108]

Rochelle-Newall, E. [25]

Rodgers, K.B. [19]

Roeckner, E. [50]

Rokityanskiy, D. [135]

Rosa, E. [95,104]

Rounsevell, M. [119]

Rounsevell, M.D.A. [118]

Rudolf, B. [87]

Ruedy, R. [68]

Running, S.R. [72]

Rustad, L.E. [16]

Sabine, C.L. [36,19]

Safir, G.R. [108]

Salameh, P.K. [37]

Saleska, S.R. [79]

Sarmiento, J.L. [34,36,19]

Sato, M. [68]

Schaphoff, S. [52,56,6,47]

Schellnhuber, H.J. [102,8]

Schienke, E. [13]

Schlesinger, W.H. [107]

Schlitzer, R. [19]

Schneider, U. [25]

Schnur, R. [50]

Scholes, R.J. [88]

Scholz, S. [11]

Schulze, E.D. [129]

Schulze, K. [41]

Schwarz, A.G. [58]

Scipal, K. [87]

Seibel, B.A. [22,23]

Senior, C.A. [64]

Shandra, J.M. [100]

Shiraishi, Y. [105]

Siegwolf, R.T.W. [55]

Silva, H. [79]

Sitch, S. [43,52,49,76,65,47,70]

Slater, R.D. [19]

Smith, B. [43,78,76,70]

Smith, J.U. [118,119]

Smith, P. [118,119]

Smith, S.D. [54]

Snitzler, K.G. [50]

Socolow, R. [33]

Sohlberg, R. [86]

Sonnerup, R.E. [38]

Soule, P. [93]

Spain, A.V. [114]

Spall, S.A. [42]

Steffen, W. [3]

Still, C.J. [77]

Strassmann, K. [50]

Strengers, Y. [101]

Sugita, S. [78]

Svirejeva-Hopkins, A. [102,8]

Svirezhev Yu, M. [90]

Sykes, M.T. [78,70]

Tan, P. [73]

Taylor, J.A. [84]

Taylor, N.K. [36]

Terbrueggen, A. [25]

Thonicke, K. [70]

Toggweiler, J.R. [36]

Totterdell, I.J. [42,19]

Townshend, J.R.G. [86]

Trenbreth, K.E. [40]

Tucker, C. [71]

Tucker, C.J. [80,72]

Umemiya, C. [9]

Urbanski, S.P. [75]

Van der Werf, G. [66]

Van der Werf, G.R. [77]

van Veen, J.A. [132]

van Vuuren, D. [41]

Venevsky, S. [70]

von Bloh, W. [50]

von Caemmerer, S. [82]

Wagner, W. [56,87]

Waley, P. [103]

Walker, K. [78]

Walker, S.J. [37]

Walker, T.W. [112]

Wallace, D.W.R. [88]

Walsh, P.J. [22,23,21]

Walz, P. [24]

Wang, Y.P. [127]

Wattenbach, M. [118,119]

Watterson, I.G. [31]

Weaver, A.J. [50]

Weirig, M.F. [19]

Weiss, R.F. [37]

Whaling, P.J. [24]

White, A. [43]

White, J.W.C. [77]

Whooley, O. [100]

Wickett, M.E. [20]

Wild, A. [131]

Williamson, J. [100]

Wofsy, S.C. [75,79]

Wood, R.A. [64]

Woodward, F.I. [43,48]

Wullschleger, S.D. [124]

Yamagata, Y. [133,135]

Yamanaka, Y. [19]

Yool, A. [19]

York, R. [104]

Yoshida, Y. [12]

Yoshikawa, C. [50]

Young-Molling, C. [43]

Zaehle, S. [118,119]

Zeng, N. [50]

Zondervan, I. [25]

Zotz, G. [55]

## Supplementary Material

Additional File 1 References to the journal articles cited in the first volume of the journal. This file is formatted for importing references into a reference database. Click here for file

Additional File 2 The list of words and wordings selected from the articles published in the first volume of the journal. Click here for file
